# Further Evidence That the Genebank Standards for Drying Orthodox Seeds May Not Be Optimal for Subsequent Seed Longevity

**DOI:** 10.1089/bio.2018.0026

**Published:** 2018-10-12

**Authors:** Katherine J. Whitehouse, Olorunnisola F. Owoborode, Olufemi O. Adebayo, Olaniyi A. Oyatomi, Amudalat B. Olaniyan, Michael T. Abberton, Fiona R. Hay

**Affiliations:** ^1^ T.T. Chang Genetic Resources Center, International Rice Research Institute, Los Baños, Philippines.; ^2^ Department of Agronomy, University of Ibadan, Ibadan, Nigeria.; ^3^ Genetic Resources Center, International Institute of Tropical Agriculture, Ibadan, Nigeria.

**Keywords:** seed longevity, genebank, seed drying, soya bean, cowpea

## Abstract

Maximizing seed longevity is important for genebanks to efficiently manage their accessions, reducing the frequency of costly regeneration cycles and the loss of genetic integrity. Research on rice seeds has shown that subsequent longevity in air-dry storage can be improved by drying seeds, which are metabolically active at harvest (moisture contents above a critical value close to 16.5%), for an initial period at a higher temperature (40°C–60°C) than that currently recommended by the current genebank standards (5°C–20°C). The aim of this study was to test whether similar benefits could be achieved in two legume species—cowpea and soya bean—by drying freshly harvested seeds, from two separate harvests, at 40°C and 35% relative humidity, for up to 8 days before equilibrium drying in a drying room (17°C and 15% relative humidity). Improvements in longevity were observed in three of the four accessions of soya bean, with the greatest improvement generally occurring after the maximum duration (8 days) at the higher temperature. However, of the five accessions of cowpea, only seeds of TVu-9698 and TVu-13209 from the first harvest, and of TVu-13193 from the second harvest, showed an improvement in longevity compared with drying following the standard protocol. A negative effect of high-temperature drying was also observed in one accession of cowpea, TVu-11980, but only in seeds harvested later in the season, 13 weeks after planting. This research not only provides evidence of the potential benefits of drying orthodox seeds at an alternative, higher, temperature instead of at the conventional lower temperature, before long-term storage, but also raises awareness of how genebanks can improve the management of their accessions.

## Introduction

The long-term preservation of the genetic diversity of orthodox species can be ensured by storing their seeds at a low temperature (−20°C) and moisture content (MC) (3%–7%) in genebanks. Breeders rely on the genetic resources to produce more high yielding varieties and/or improve their resistance to a wider range of biotic/abiotic stresses.^1^ Therefore, it is critical that genebanks effectively manage accessions, by monitoring their viability at regular intervals and regenerating them when germination falls.^2–5^

Recommendations for the management of genebank accessions emphasize the importance of initial seed drying to extend the subsequent storage longevity.^2,3,4,6^ It has been reported that there is an upper temperature limit for safe drying, which varies between species and depends on the MC of the seeds—as seeds dry, the maximum safe drying temperature increases.^7^ Consequently, the values of safe drying temperatures for seed drying reported in the literature are not consistent. For example, it has been claimed that a safe drying temperature for rice seeds is 48.9°C and 60°C when seeds are at an MC >20% and <15%, respectively,^8^ whereas onion seeds are particularly vulnerable, and are recommended not to be dried at temperatures exceeding 20.1°C when seeds are at an MC >20%.^9^ More generally, a cooler limit of 45°C and 35°C has been suggested when drying cereal and vegetable seeds, respectively.^10^

In 1994, The Genebank Standards were published and recommended to dry seeds at 10°C–20°C and 10%–15% relative humidity (RH).^6^ More recently, these drying standards were modified, combining a lower temperature (5°C–20°C) and broader humidity (10%–25% RH) range.^2^ It is important to note that these standards were developed based on their suitability to dry mature seeds of a very diverse range of species (all crops and wild relatives with orthodox seeds, from across the globe) to a low MC for storage and, therefore, are neither species specific nor dependant on initial seed moisture. However, as mature seeds at high temperatures are likely to be more sensitive to damage,^7,11^ especially during the later stages of drying when evaporative cooling can no longer suppress seed temperature,^3^ a low temperature combined with a low humidity was adopted.^2,6^

Evidence from previous studies on rice, both cultivated^12–15^ and wild,^16^ suggests that these conditions, in particular the lower drying temperature of the modified standards, are not optimum for subsequent rice seed longevity when seeds are harvested at an MC close to 16.5%.^12–14^ Similarly, research on pea^17^ and soya bean^18^ has shown that the physiological quality (vigor and viability) of seeds can be maintained following drying at 40°C, but declines at temperatures ≥50°C.

The aim of this study was to evaluate whether similar benefits to longevity (as seen in rice) could be achieved in seeds of cowpea and soya bean (two of the mandate crops conserved at the International Institute of Tropical Agriculture [IITA]) when they are dried for an initial period at a higher temperature (40°C) before equilibrium drying at a cooler temperature (17°C which complies with the current genebank standards). Based on the response seen in rice,^12–16^ the aim was to dry seeds as close to 45°C as possible; however, due to equipment constraints, a temperature of 40°C was used.

## Materials and Methods

### Plant material

Seeds of four soya bean accessions (TGm-22, TGm-891, TGm-1013, and TGm-1014) and five cowpea accessions (TVu-8851,^19^ TVu-9698,^20^ TVu-11980,^21^ TVu-13193; and TVu-13209), of varying seed coat thickness, were sampled from the active collection of the IITA genebank. Seeds from both crops were grown according to the normal regeneration procedure at IITA, with standard production practices and plant protection measures.^22^ Seeds were sown in two 100 m^2^ plots on November 10, 2016, and harvested, when seeds had reached an MC between 13% and 18% (∼12 and 13 weeks after planting), on two separate occasions on January 31, 2017 (Harvest A) and February 6, 2017 (Harvest B). For each of the harvests, seeds were randomly sampled across the two plots.

Immediately after each harvest, a small subsample of pods, from each accession, enough to fill the precalibrated AgriPro 6095 MC analyzer (SINAR™), was hand-threshed and the seed harvest MC (fresh weight basis) determined. The freshly harvested pods from each accession were bagged in bulk and transferred to the fumigation chamber (25°C–27°C and 50% RH) where they were exposed to 57% aluminum phosphide (Phostoxin) for ∼24 hours. Following fumigation, the MC of a sample of seeds from threshed pods of each accession was measured, as before, before discarding.

### Seed drying

For each accession × harvest, with the exception of accession TVu-9698 from harvest B, the remaining fumigated pods were divided into five (equating to the five drying treatments) 1 kg (cowpea) or 1.4 kg (soya bean) samples and placed into mesh bags. Due to the low yield of accession TVu-9698, there were insufficient seeds to carry out two full sets (harvests A and B) of drying treatments, and therefore, only two out of the four high-temperature treatments (days 2 and 8 of drying), plus the drying room (DR) control, were carried out for harvest B. A sample from each accession was immediately placed into the DR and maintained at 17°C/15% RH for 7 days (intact pod drying) before measuring seed MC, following the same method as before. Pods were then threshed and the extracted seeds were given a second 24-hour cycle of fumigation before returning to the DR for 7 days, to reach equilibrium (seed drying). The remaining four samples per accession (or two for accession TVu-9698 from harvest B), instead of immediate drying in the DR, underwent an initial period of high-temperature drying in a chamber maintained at 40°C and 35% RH (intact pod drying). After 2, 4, 6 and 8 days, a sample was removed and seed MC determined, as before. The remaining pods from this sample were transferred to the DR for 7 days (intact pod drying) before measuring the MC once again. The pods were then threshed and seeds fumigated for 24 hours before final drying in the DR (seed drying).

After all seed samples (control and high-temperature treatments) had undergone a total of 14 days in the DR, the final MC was determined. The seed lots were then sent for manual cleaning where any empty, damaged, and/or diseased seeds were removed. Clean seeds were then sealed inside labeled aluminum foil packets (accession × harvest × treatment combination) and stored in the medium-term storage facility (5°C) for 7 days, until the storage experiments began.

### Seed storage

Seed lots (accession × harvest × treatment combination) were removed from the medium-term storage facility and equilibrated at room temperature before opening. Hard coated seeds (accessions TVu-9698, TVu-11980, and TGm-891) were scarified using a surgical blade to allow the uptake of moisture. Each sample was then transferred to a cloth bag and placed over water in a Percival incubator set at 25°C until they had reached 60% RH (equating to 11.1% and 9.0% MC for cowpea and soya bean, respectively [calculated using the Seed Information Database; SID^23^]). The MC was estimated daily by monitoring the change in weight. Once the desired weight had been achieved, the seeds were sealed inside aluminum foil packets and left to equilibrate at room temperature for 24 hours to ensure even moisture distribution.

The seeds from each packet were divided into either 19 (cowpea) or 18 (soya bean), 15 g subsamples and sealed inside smaller aluminum foil packets. A subsample was immediately used to estimate initial ability to germinate, before experimental storage, and the remaining subsamples were placed in a Percival incubator at 45°C for a maximum of 42 and 72 days for cowpea and soya bean, respectively. A sample was removed at regular intervals during storage for germination testing.

### Seed germination

The 60 seeds from each packet were subdivided for sowing onto Seedburo K-22 germination paper and wetted with distilled water, in plastic germination boxes or germination plates (20 seeds per box/plate). Cowpea seeds were mixed with a small amount of Mancozeb powder (antifungal agent) before being sown. Seeds were then transferred to an incubation room at 25°C–30°C and 68% RH for 10 days before the total number of germinated seeds was recorded.

### Statistical analyses

For each accession × treatment combination, change in the ability to germinate after different periods of experimental storage was analyzed by probit analysis, using GenStat for Windows, Version 18 (VSN International Ltd., Hemel Hempsted, United Kingdom), thereby fitting the following viability equation^24^:

\begin{align*}
v = {K_i} - \left(   {p / \sigma } \right) , \tag{1}
\end{align*}

where *v* is the viability (NED) after *p* days in storage (45°C/60% RH), *K_i_* is the initial viability (NED), and *σ* (days) is the standard deviation of the normal distribution of seed deaths in time. The time for viability to decline to 50% (*p*_50_) is a product of *K*_i_ and *σ* (*p*_50_ = *K_i_* × *σ*), and was used as a measure of longevity. For those seed lots (accession × harvest × drying treatment) that showed a reduced initial viability, the “control mortality” parameter (“immunity” in GenStat) was included in the probit analysis to estimate the proportion of “nonresponding” seeds within the seed lot.^25^ Probit analysis was carried out for all seed lots within an accession simultaneously, fitting the full model (different estimates for each parameter) and reduced models (one or more parameter constrained to the same value for different seed lots). An approximate *F*-test was used to determine the best model.

## Results

### Seed drying

The harvest MC of four out of the five cowpea accessions, with the exception of accession TVu-11980, was higher when seeds were harvested later in the season (harvest B), at 13 weeks after planting, compared with when seeds were harvested a week earlier (harvest A) (Table 1). The harvest MC of accessions ranged between 10.7% and 13.3% in harvest A and between 7.6% and 17.2% in harvest B. Accessions TVu-9698 and TVu-11980 showed the highest and the lowest MC, respectively, in both harvests. All seed lots lost moisture over time, irrespective of the timing of harvest and/or drying treatment, and reached a final MC between 6% and 8.7% following 14 days in the DR. Generally, seed lots that underwent an initial period of drying at 40°C/35% RH lost the most moisture during the first 2 days, followed by a gradual decline until equilibrium was reached. Seed lots from harvest A reached their lowest MC after 6 days of drying at 40°C/35% RH compared with seed lots from harvest B, which reached their lowest MC after 8 days of drying.

**Table 1.** Moisture Content (% Fresh Weight) of Seeds from Five Accessions of Cowpea and Four Accessions of Soya Bean Harvested on January 31, 2017 (Harvest A), and February 6, 2017 (Harvest B), Which Were Either Immediately Dried After Harvest in the Genebank Drying Room (17°C/15% Relative Humidity) or Initially Dried at 40°C/35% Relative Humidity for 2, 4, 6, or 8 Days Before Equilibrium Drying (Total 14 Days) in the Drying Room

*Cowpea*										
	*TVu-8851*		*TVu-9698*^a^		*TVu-11980*^a^		*TVu-13193*		*TVu-13209*	
*Harvest*	*A*	*B*	*A*	*B*	*A*	*B*	*A*	*B*	*A*	*B*
Harvest MC	12.0	15.9	13.3	17.2	10.7	7.6	12.2	13.1	12.5	15.0
High-temperature drying										
40°C/35% RH (2d)	9.8	10.2	6.7	6.7	9.0	9.7	8.9	8.5	9.3	9.1
40°C/35% RH (4d)	9.4	6.8	8.1	—	10.8	6.0	8.6	7.8	8.5	9.7
40°C/35% RH (6d)	7.5	8.2	6.3	—	6.4	7.3	7.5	6.7	7.6	7.7
40°C/35% RH (8d)	7.5	8.0	6.4	6.0	6.3	6.0	7.5	6.0	7.6	7.3
→DR (7d)	7.4	6.9	6.5	6.1	7.1	7.6	7.2	7.5	7.5	7.8
→DR (14d)	7.5	7.6	6.0	6.0	7.0	8.1	7.4	7.0	7.3	7.9
Control										
DR (7d)	8.7	10.4	6.8	8.2	9.3	8.9	8.4	8.0	9.6	8.9
DR (14d)	8.0	7.3	6.0	7.4	8.7	7.5	8.6	7.1	7.8	8.2

*Soya bean*								
	*TGm-22*		*TGm-891*^a^		*TGm-1013*		*TGm-1014*	
*Harvest*	*A*	*B*	*A*	*B*	*A*	*B*	*A*	*B*
Harvest MC	17.6	15.0	11.8	15.3	13.7	12.9	26.8	23.1
High-temperature drying								
40°C/35% RH (2d)	8.6	7.0	8.5	7.5	9.5	10.2	14.8	8.3
40°C/35% RH (4d)	8.7	6.5	8.6	6.2	9.7	7.7	8.7	6.4
40°C/35% RH (6d)	8.5	7.5	8.4	7.2	9.3	7.4	8.6	6.6
40°C/35% RH (8d)	8.6	7.8	8.5	6.9	9.6	6.6	8.6	7.4
→DR (7d)	7.7	7.5	7.5	7.3	7.9	7.6	7.0	7.7
→DR (14d)	7.6	7.4	7.9	7.6	7.7	7.7	7.9	7.9
Control								
DR (7d)	7.1	7.7	7.0	8.4	7.1	9.1	6.9	9.3
DR (14d)	8.5	8.0	8.4	8.1	8.1	8.1	8.3	7.5

The MC was determined, nondestructively, using a seed MC analyzer (SINAR AGRIPO). Due to the low yield of accession TVu-9698, there were insufficient seeds from harvest B to carry out the full set of drying treatments. Therefore, only two out of the four high-temperature treatments (2 and 8d), plus the DR control, were carried out.

^a^ Hard-coated seeds.

d, days; DR, drying room; MC, moisture content.

After the cowpea seeds were transferred to the DR, the MC of most seed lots, although they fluctuated slightly, generally reached their lowest recorded MC after 14 days (Table 1). Furthermore, in general, seeds that were exposed to an initial period of high-temperature drying reached a lower final MC (after 14 days in the DR) compared with when seeds were immediately placed in the DR. However, seeds of accession TVu-11980 from both harvests and seeds of accession TVu-13191 and TVu-13209 from harvest B regained moisture in the DR, which led them to be at a higher final MC than what was achieved after 8 days at 40°C.

The harvest MC varied between accessions of soya bean, with the accession with the largest seeds (TGm-1014) showing the highest MC in both harvests (26.8% and 23.1% in harvest A and harvest B, respectively) and the accession with the hardest seed coat (TGm-891) showing the lowest MC (11.8%), at least in harvest A (Table 1). With the exception of accession TGm-1013, seeds harvested earlier in the season (harvest A), 12 weeks after planting, showed a higher MC compared with seeds harvested a week later (harvest B)—the opposite to what was observed in cowpea. However, as in cowpea, soya bean seeds that were dried for an initial period at 40°C/35% RH lost the most moisture during the first 2 days of drying and, as expected, seeds that were at a higher MC at harvest took longer to reach their lowest recorded MC (after 6 days of drying at 40°C) compared with lower MC seeds; they reached their lowest MC after only 4 days. Furthermore, the seed lots from harvest B, with the exception of accession TGm-22, appeared to regain moisture after being transferred for final drying in the DR compared with seed lots from harvest A, which all continued to dry in the DR, and reached their lowest recorded MC after 14 days.

For all the soya bean accessions, from both harvests, seeds that underwent an initial period of high-temperature drying before being transferred for final drying in the DR reached a lower final MC (after 14 days of drying in the DR) compared with seeds that were immediately placed in the DR (Table 1). However, in most seed lots, DR-dried seeds appeared to regain moisture during drying in the DR, with their lowest recorded MC generally being after only 7 days of drying.

### Cowpea seed longevity

**FIG. 1.**
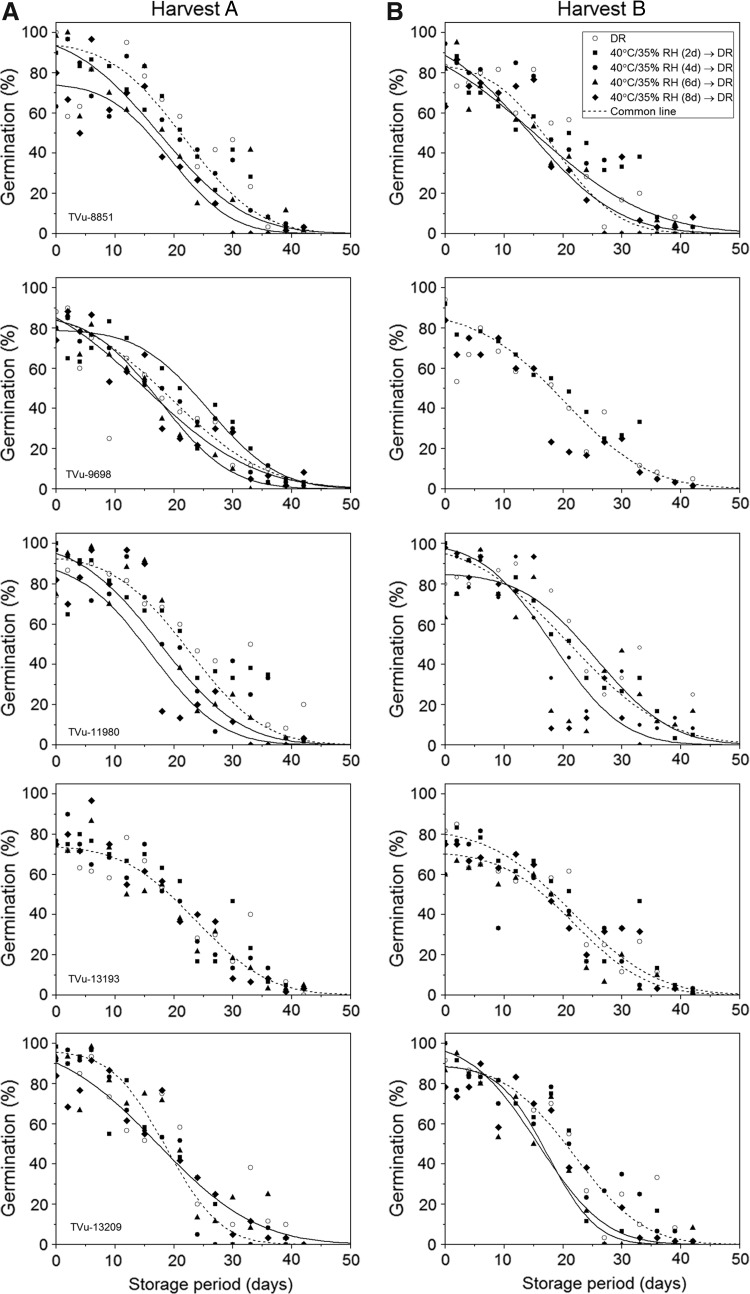
Ability to germinate when tested during experimental storage at 45°C and 60% RH for seeds of five accessions of cowpea harvested on January 31, 2017 (Harvest **A**), and February 6, 2017 (Harvest **B**), which were immediately dried after harvest in the DR (17°C/15% RH) or initially at 40°C/35% RH for 2, 4, 6, or 8 days before equilibrium drying in the dryroom. The viability model^24^ was fitted to the data with or without parameter constraints; the results shown are for the model with the fewest parameters that could be fitted without a significant increase in the residual deviance compared with the best-fit model (Table 2). The *dashed lines* correspond to treatments that could be constrained to a single curve (*p* > 0.05). For those seed lots that showed a reduced initial viability (<100%), an additional parameter was applied to determine the proportion of responding seeds within the population.^25^ DR, dryroom; RH, relative humidity.

Seed longevity varied between accessions of cowpea and, in some cases, between treatments within each accession (Fig. 1; Table 2). Although dormancy was not observed, there were many instances where drying, either in the DR or at the higher temperature, reduced the proportion of responding seeds within the population (proportion of nonresponders; Table 2). This asymmetry in the survival curve did not appear to be linked to any particular drying treatment. The longevity (*p*_50_) of seeds when dried following the standard protocol at IITA (DR) ranged between 17.2 and 23.7 days and between 18.6 and 25.7 days for harvests A and B, respectively, with seeds harvested later in the season generally showing the higher estimates in longevity for each accession.

**Table 2.** Results of Fitting the Viability Model for Samples of Cowpea Harvested on January 31, 2017 (Harvest A), and February 6, 2017 (Harvest B), Which Were Immediately Dried After Harvest in the Dryroom (17°C/15% Relative Humidity) or Initially at 40°C/35% Relative Humidity for 2, 4, 6, or 8 Days Before Equilibrium Drying in the Dryroom

*Treatment*	*Model*	*Proportion of nonresponders (s.e.)*	K_i_*(s.e.) (NED)*	σ^−^*^1^ (s.e.) (days^−1^)*	p*_50_ (s.e.) (days)*
Harvest A					
TVu-8851	*K_i_* and *σ* constrained within 40°C/35% RH (2d) and (4d) → DR and DR				
40°C/35% RH (2d) → DR		0.050 (0.020)	2.20 (0.17)	0.10 (0.01)	21.8 (0.61)
40°C/35% RH (4d) → DR		0.050 (0.020)	2.20 (0.17)	0.10 (0.01)	21.8 (0.61)
40°C/35% RH (6d) → DR		—	1.50 (0.12)	0.09 (0.01)	17.4 (0.73)
40°C/35% RH (8d) → DR		0.254 (0.046)	2.34 (0.39)	0.12 (0.01)	19.3 (1.32)
DR		0.050 (0.020)	2.20 (0.17)	0.10 (0.01)	21.8 (0.61)
TVu-9698	*K_i_* and *σ* constrained within 40°C/35% RH (4d) → DR and DR and *σ* constrained within 40°C/35% RH (2d) and (6d) → DR				
40°C/35% RH (2d) → DR		0.211 (0.034)	2.76 (0.30)	0.11 (0.01)	25.9 (1.10)
40°C/35% RH (4d) → DR		0.101 (0.049)	1.52 (0.24)	0.08 (0.01)	19.5 (1.42)
40°C/35% RH (6d) → DR		0.136 (0.044)	1.89 (0.25)	0.11 (0.01)	17.7 (1.12)
40°C/35% RH (8d) → DR		—	1.04 (0.11)	0.07 (0.00)	15.1 (0.85)
DR		0.101 (0.049)	1.52 (0.24)	0.08 (0.01)	19.5 (1.42)
TVu-11980	*K_i_* and *σ* constrained within 40°C/35% RH (2d) and (6d) → DR and DR				
40°C/35% RH (2d) → DR		0.065 (0.021)	2.28 (0.19)	0.10 (0.01)	22.6 (0.61)
40°C/35% RH (4d) → DR		—	1.65 (0.13)	0.09 (0.01)	17.7 (0.72)
40°C/35% RH (6d) → DR		0.065 (0.021)	2.28 (0.19)	0.10 (0.01)	22.6 (0.61)
40°C/35% RH (8d) → DR		0.095 (0.068)	1.72 (0.42)	0.11 (0.01)	16.1 (2.10)
DR		0.065 (0.021)	2.28 (0.19)	0.10 (0.01)	22.6 (0.61)
TVu-13193	*K_i_* and *σ* constrained within all treatments				
40°C/35% RH (2d) → DR		0.258 (0.017)	2.42 (0.18)	0.10 (0.01)	23.7 (0.59)
40°C/35% RH (4d) → DR					
40°C/35% RH (6d) → DR					
40°C/35% RH (8d) → DR					
DR					
TVu-13209					
40°C/35% RH (2d) → DR	*K_i_* and *σ* constrained within 40°C/35% RH (2d), (4d), and (6d) → DR	0.040 (0.013)	2.54 (0.17)	0.14 (0.01)	18.7 (0.38)
40°C/35% RH (4d) → DR		0.040 (0.013)	2.54 (0.17)	0.14 (0.01)	18.7 (0.38)
40°C/35% RH (6d) → DR		0.040 (0.013)	2.54 (0.17)	0.14 (0.01)	18.7 (0.38)
40°C/35% RH (8d) → DR		0.175 (0.057)	1.99 (0.39)	0.10 (0.01)	20.6 (1.70)
DR		—	1.30 (0.10)	0.08 (0.00)	17.2 (0.76)
Harvest B					
TVu-8851	*K_i_* and *σ* constrained within 40°C/35% RH (4d) and (6d) → DR and DR				
40°C/35% RH (2d) → DR		—	0.97 (0.10)	0.06 (0.00)	15.1 (0.94)
40°C/35% RH (4d) → DR		0.156 (0.028)	2.19 (0.26)	0.12 (0.01)	18.6 (0.74)
40°C/35% RH (6d) → DR		0.156 (0.028)	2.19 (0.26)	0.12 (0.01)	18.6 (0.74)
40°C/35% RH (8d) → DR		—	1.21 (0.13)	0.08 (0.01)	14.4 (0.83)
DR			2.19 (0.26)	0.12 (0.01)	18.6 (0.74)
TVu-9698	*K_i_* and *σ* constrained within all treatments				
40°C/35% RH (2d) → DR		0.124 (0.043)	1.75 (0.22)	0.09 (0.01)	20.1 (1.15)
40°C/35% RH (8d) → DR					
DR					
TVu-11980	*K_i_* and *σ* constrained within 40°C/35% RH (2d), (4d), and (6d) → DR				
40°C/35% RH (2d) → DR		—	1.64 (0.07)	0.08 (0.00)	21.2 (0.45)
40°C/35% RH (4d) → DR		—	1.64 (0.07)	0.08 (0.00)	21.2 (0.45)
40°C/35% RH (6d) → DR		—	1.64 (0.07)	0.08 (0.00)	21.2 (0.45)
40°C/35% RH (8d) → DR		—	1.96 (0.14)	0.11 (0.01)	18.2 (0.68)
DR		0.149 (0.030)	2.57 (0.36)	0.10 (0.01)	25.7 (1.06)
TVu-13193	*K_i_* and *σ* constrained within 40°C/35% RH (2d) and (4d) → DR and DR and within 40°C/35% RH (6d) and (8d)				
40°C/35% RH (2d) → DR		0.176 (0.035)	1.91 (0.22)	0.09 (0.01)	21.6 (1.08)
40°C/35% RH (4d) → DR		0.176 (0.035)	1.91 (0.22)	0.09 (0.01)	21.6 (1.08)
40°C/35% RH (6d) → DR		0.290 (0.031)	2.26 (0.32)	0.10 (0.01)	21.9 (1.25)
40°C/35% RH (8d) → DR		0.290 (0.031)	2.26 (0.32)	0.10 (0.01)	21.9 (1.25)
DR		0.176 (0.035)	1.91 (0.22)	0.09 (0.01)	21.6 (1.08)
TVu-13209	*K_i_* and *σ* constrained within 40°C (4d) and (8d) → DR and DR				
40°C (2d) → DR		—	1.76 (0.11)	0.11 (0.01)	15.8 (0.62)
40°C (4d) → DR		0.105 (0.025)	2.32 (0.20)	0.11 (0.01)	21.8 (0.69)
40°C (6d) → DR		0.114 (0.028)	2.67 (0.44)	0.15 (0.02)	17.4 (0.86)
40°C (8d) → DR		0.105 (0.025)	2.32 (0.20)	0.11 (0.01)	21.8 (0.69)
DR		0.105 (0.025)	2.32 (0.20)	0.11 (0.01)	21.8 (0.69)

Source: Ellis and Roberts, 1980.^24^

The parameters shown are for the simplest model that could be fitted without a significant (*p* < 0.05) increase in the residual deviance compared with the best fit model. Due to the low yield of accession TVu-9698, there were insufficient seeds from harvest B to carry out the full set of drying treatments. Therefore, storage experiments were only carried out for two of the four high-temperature treatments (2 and 8 days), plus the DR control. For those samples that showed a reduced initial viability, the “control mortality” was applied to determine the proportion of responding seeds within the population.^13^

s.e., standard error.

An improvement in longevity when drying seeds for an initial period at 40°C/35% RH compared with solely drying in the DR was only seen in accessions TVu-9698 and TVu-13209, from harvest A, which showed the greatest value of *p*_50_ after 2 and 8 days, respectively, and in accession TVu-13193, from harvest B, which showed the greatest value of *p*_50_ after 6 days of drying, and was maintained thereafter (Fig. 1; Table 2). The remaining accessions from each harvest, other than accession TVu-11980 from harvest B, which showed an overall negative effect of high-temperature drying on subsequent seed longevity, drying for an initial period at 40°C/35% RH did not improve the longevity compared with drying in the DR throughout (i.e., *K*_i_ and σ could be constrained [*p* < 0.05] for 1 or more of the high-temperature treatments (2–8 days) and the DR). Out of these six accessions × harvest, with the exception of TVu-13193 from harvest A and TVu-9698 from harvest B where there was no significant difference in the longevity between any of the drying treatments, the response of seeds to high-temperature drying was variable depending on the duration of drying; generally, the seeds reached the same longevity, as achieved when drying in the DR, after 2 or 4 days of drying and declined thereafter (Table 2). Furthermore, there did not appear to be any relationship between the duration of high-temperature drying, which produced the lowest estimates of *p*_50_, and its effect on *K_i_*, *σ*, and/or the proportion of non-responders (Table 2). To conclude, on average (7 out of the 10 seed samples tested), drying cowpea seeds for an initial period at a high temperature did not improve their subsequent longevity compared with drying following the standard protocol at IITA.

### Soya bean seed longevity

**FIG. 2.**
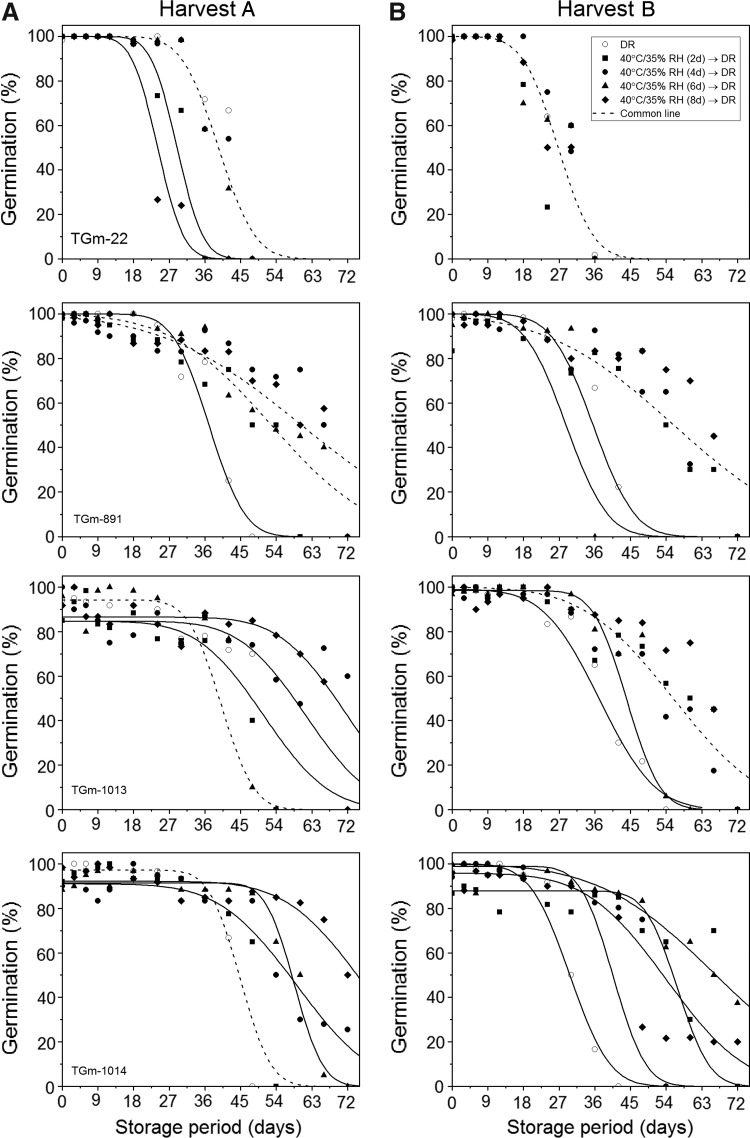
Ability to germinate when tested during experimental storage at 45°C and 60% RH for seeds of four accessions of soya bean harvested on January 31, 2017 (Harvest **A**), and February 6, 2017 (Harvest **B**), which were immediately dried after harvest in the DR (17°C/15% RH) or initially at 40°C/35% RH for 2, 4, 6, or 8 days before equilibrium drying in the dryroom. The viability model^21^ was fitted to the data with or without parameter constraints; the results shown are for the model with the fewest parameters that could be fitted without a significant increase in the residual deviance compared with the best-fit model (Table 3). The *dashed lines* correspond to treatments that could be constrained to a single curve (*p* > 0.05). For those seed lots that showed a reduced initial viability (<100%), an additional parameter was applied to determine the proportion of responding seeds within the population.^26^

As observed in cowpea, the longevity of soya bean seeds varied both between accessions and between harvests, with higher estimates of DR *p*_50_ seen in seeds from harvest A compared with seeds from harvest B (Fig. 2; Table 3). The accessions showing the greatest longevity (*p*_50_) following drying in the DR were TGm-1014 and TGm-1013 for harvests A and B, respectively. Differences in longevity (resulting from differences in *K_i_*, *σ*, or both (Table 3)) were also observed, in some cases, among the different drying treatments within accessions. Despite these differences, with the exception of accession TGm-22, at least one of the high-temperature treatments (period of drying at 40°C/35% RH) resulted in an improvement in longevity (*p*_50_) compared with drying in the DR. In all instances, with the exception of accession TGm-1014 from harvest B, the highest estimates of *p*_50_ were observed in seed lots dried for 8 days at 40°C/35% RH. Although drying at this higher temperature, for up to 8 days, resulted in lower values of *K_i_* (initial viability) compared with the DR control, seeds lost viability at a slower rate. In both harvests, accession TGm-1014 showed the greatest longevity following drying at 40°C/35% RH after 8 (harvest A) and 6 days (harvest B), with values of *p*_50_ estimated at 76 and 66.6 days for harvests A and B, respectively. Accession TGm-22, from both harvests, was the only accession where drying for an initial period at 40°C/35% RH did not improve subsequent storage longevity compared with the DR control. In harvest A, there was no significant difference (*p* < 0.05) in the longevity between seeds dried at 40°C/35% RH for 4 and 6 days and the DR control, with seeds dried for 8 days showing the lowest value of *K_i_* and estimate of *p*_50_ (Table 3). On the other hand, in harvest B, all drying treatments could be constrained to a common line, that is, there was no significant difference in longevity between any of the drying treatments.

**Table 3.** Results of Fitting the Viability Model for Samples of Soya Bean Harvested on January 31, 2017 (Harvest A), and February 6, 2017 (Harvest B), Which Were Immediately Dried After Harvest in the Dryroom (17°C/15% Relative Humidity) or Initially at 40°C/35% Relative Humidity for 2, 4, 6, or 8 Days before Equilibrium Drying in the Dryroom

*Treatment*	*Model*	*Proportion of nonresponders (s.e.)*	K_i_*(s.e.) (NED)*	σ*^−1^ (s.e.) (days^−1^)*	p*_50_ (s.e.) (days)*
Harvest A					
TGm-22	*K_i_* and *σ^−^*^1^ constrained within 40°C/35% RH (4d) and (6d) → DR and DR and *σ^−^*^1^ constrained within 40°C/35% RH (2d) and (8d) → DR				
40°C/35% RH (2d) → DR		—	5.59 (0.39)	0.19 (0.01)	29.0 (0.55)
40°C/35% RH (4d) → DR		—	5.36 (0.29)	0.14 (0.01)	39.4 (0.39)
40°C/35% RH (6d) → DR		—	5.36 (0.29)	0.14 (0.01)	39.4 (0.39)
40°C/35% RH (8d) → DR		—	4.65 (0.33)	0.19 (0.01)	24.1 (0.54)
DR		—	5.36 (0.29)	0.14 (0.01)	39.4 (0.39)
TGm-891	*K_i_* and *σ^−^*^1^ constrained within 40°C/35% RH (2d) and (6d) → DR and within 40°C/35% RH (4d) and (8d) → DR				
40°C/35% RH (2d) → DR		—	2.64 (0.11)	0.05 (0.00)	50.5 (0.89)
40°C/35% RH (4d) → DR		—	2.17 (0.09)	0.04 (0.00)	60.9 (1.40)
40°C/35% RH (6d) → DR		—	2.64 (0.11)	0.05 (0.00)	50.5 (0.89)
40°C/35% RH (8d) → DR		—	2.17 (0.09)	0.04 (0.00)	60.9 (1.40)
DR		—	5.18 (0.45)	0.14 (0.01)	37.0 (0.66)
TGm-1013	*K_i_* and *σ^−^*^1^ constrained within 40°C/35% RH (6d) → DR and DR and *σ^−^*^1^ constrained within 40°C/35% RH (2d), (4d), and (8d) → DR				
40°C/35% RH (2d) → DR		0.153 (0.022)	3.87 (0.66)	0.08 (0.01)	50.8 (2.71)
40°C/35% RH (4d) → DR		0.153 (0.023)	4.68 (0.82)	0.08 (0.01)	61.4 (2.47)
40°C/35% RH (6d) → DR		0.058 (0.009)	6.11 (0.57)	0.15 (0.01)	40.1 (0.73)
40°C/35% RH (8d) → DR		0.135 (0.017)	5.45 (0.92)	0.08 (0.01)	71.5 (3.11)
DR		0.058 (0.009)	6.11 (0.57)	0.15 (0.01)	40.1 (0.73)
TGm-1014	*K_i_* and *σ^−^*^1^ constrained within 40°C/35% RH (2d) → DR and DR and *σ^−^*^1^ constrained within 40°C/35% RH (4d) and (8d) → DR				
40°C/35% RH (2d) → DR		0.028 (0.007)	7.30 (0.85)	0.16 (0.02)	44.9 (0.62)
40°C/35% RH (4d) → DR		0.090 (0.014)	3.86 (0.43)	0.07 (0.01)	59.1 (1.36)
40°C/35% RH (6d) → DR		0.085 (0.011)	10.22 (1.36)	0.17 (0.02)	58.6 (0.73)
40°C/35% RH (8d) → DR		0.078 (0.01)	4.96 (0.49)	0.07 (0.01)	76.0 (2.22)
DR		0.028 (0.007)	7.30 (0.85)	0.16 (0.02)	44.9 (0.62)
Harvest B					
TGm-22	*K_i_* and *σ^−^*^1^ constrained within all treatments				
40°C/35% RH (2d) → DR		—	3.79 (0.14)	0.14 (0.01)	26.7 (0.29)
40°C/35% RH (4d) → DR		—			
40°C/35% RH (6d) → DR		—			
40°C/35% RH (8d) → DR		—			
DR		—			
TGm-891	*K_i_* and *σ^−^*^1^ constrained within 40°C/35% RH (2d), (4d), and (8d) → DR and *σ^−^*^1^ constrained within 40°C/35% RH (6d) → DR and DR				
40°C/35% RH (2d) → DR		—	2.22 (0.07)	0.04 (0.00)	56.0 (0.92)
40°C/35% RH (4d) → DR		—	2.22 (0.07)	0.04 (0.00)	56.0 (0.92)
40°C/35% RH (6d) → DR			3.49 (0.01)	0.12 (0.01)	28.7 (0.10)
40°C/35% RH (8d) → DR		—	2.22 (0.07)	0.04 (0.00)	56.0 (0.92)
DR			4.31 (0.28)	0.12 (0.01)	35.6 (0.70)
TGm-1013	*K_i_* and *σ^−^*^1^ constrained within 40°C/35% RH (2d), (4d), and (8d) → DR				
40°C/35% RH (2d) → DR		—	3.12 (0.13)	0.06 (0.00)	55.8 (0.74)
40°C/35% RH (4d) → DR		—	3.12 (0.13)	0.06 (0.00)	55.8 (0.74)
40°C/35% RH (6d) → DR		0.015 (0.006)	6.61 (0.69)	0.15 (0.02)	44.1 (0.82)
40°C/35% RH (8d) → DR		—	3.12 (0.13)	0.06 (0.00)	55.8 (0.74)
DR		0.010 (0.009)	3.34 (0.36)	0.09 (0.01)	37.2 (1.03)
TGm-1014	*σ^−^*^1^ constrained within 40°C/35% RH (2d) and (4d) → DR				
40°C/35% RH (2d) → DR		0.122 (0.015)	8.79 (1.03)	0.15 (0.02)	56.9 (0.88)
40°C/35% RH (4d) → DR		0.012 (0.006)	6.28 (0.66)	0.15 (0.02)	40.6 (1.20)
40°C/35% RH (6d) → DR		—	2.88 (0.21)	0.04 (0.00)	66.6 (2.13)
40°C/35% RH (8d) → DR		0.042 (0.013)	3.41 (0.38)	0.06 (0.01)	54.1 (1.52)
DR		—	3.72 (0.30)	0.13 (0.01)	29.7 (0.69)

Ellis and Roberts, 1980.^24^

The parameters shown are for the simplest model that could be fitted without a significant (*p* < 0.05) increase in the residual deviance compared with the best fit model. For those samples that showed a reduced initial viability, the “control mortality” was applied to determine the proportion of responding seeds within the population.^25^

## Discussion

Cowpea and soya bean are valuable and economically important agricultural commodities, helping to sustain millions of people within Africa and Asia. The IITA Genetic Resources Center currently holds the largest and most diverse collection of cowpea (>15,000 accessions), and nearly 5000 accessions of soya bean, which are made available for food and agricultural research across the globe. Seed longevity is an important agronomic factor, which, if not maximized, can lead to loss in seed vigor and viability during storage, thus negatively impacting seedling establishment and the overall yield. It is important for genebanks to predict the longevity of their accessions as an unexpected loss in viability will incur more frequent regeneration, which is costly economically and risky genetically.^4,26,27^ This is particularly problematic for species that are inherently short-lived as they already have short regeneration intervals. In comparison with cowpea (oil content of 1%),^28^ which has a predicted longevity (*σ*) of 301 years under medium-term storage (6.1% MC and 5°C) at IITA (estimated using the SID),^23^ soya bean (oil content of 21%^29^) seeds are relatively short-lived with an estimated longevity of only 92 years based on the viability constants given in Ellis et al.^30^ and Dickie et al.^31^.

As in cultivated rice, longevity in legumes increases during seed development, particularly during seed maturation.^32,33^ Previous research on rice has provided evidence that seed quality^15^ and subsequent seed longevity^12–14,16^ can be improved *ex planta* by drying seeds immediately after harvest, which are still within the maturation drying phase of seed development (MCs close to 16.5%),^12–14^ for an initial period at a high temperature (45°C–60°C) before final drying at a lower temperature and humidity, as recommended in the Genebank Standards.^2^ The results of this study have major implications for genebanks globally as it has shown that such benefits are not limited to rice, and potentially, a two-stage drying procedure could also improve storage longevity of other orthodox species.

In general, cowpea accessions did not show the same, consistent, beneficial response to high-temperature drying as observed in soya bean, with only three seed lots (from accessions TVu-9698, TVu-13209, and TVu-13193) showing an increase in longevity, all after different durations of drying, compared with the DR control (Fig. 2 and Table 3). Furthermore, despite this positive response, the improvement in longevity was relatively low, with an improvement in longevity by 33%, 20%, and 1% for accessions TVu-9698, TVu-13209, and TVu-13193, respectively. In soya bean, however, high-temperature drying has the potential to improve the longevity of accessions by more than 100%, as the value of *p*_50_ in seeds of accession TGm-1014 more than doubled following 6 days of drying at 40°C/35% RH (66.6 days) compared with the DR control (29.7 days) (Table 3). In fact, TGm-22 was the only accession that did not show any improvement in longevity in response to high-temperature drying, nor a negative effect, with all other accessions showing the greatest response following 6 or 8 days of drying at 40°C/35% RH. This is consistent with the previous research carried out by Whitehouse et al.^12–14^ who also reported, in rice, that while most of the drying occurred after only 1–2 days at the higher temperature, longevity continued to improve with drying duration.

Although in most cases in this study, drying at the higher temperature for 8 days tended to reduce the initial viability of the seed lot (lower *K_i_* value) compared with the DR control, the seeds remained viable for longer during storage (lower value of *σ*^−1^). This implies that high-temperature exposure may have been detrimental to short-lived seeds within the population, which were already on the cusp of losing viability, but was beneficial to longer-lived seeds, enabling them to continue to accrue longevity. It has recently been reported in soya bean that an increase in longevity during the maturation drying phase is associated with the increase in transcription factors and gene expression involved in encoding protective proteins (e.g., heat-shock proteins and chaperones) and other protective mechanisms such as sugar metabolism (raffinose family oligosaccharides/sucrose ratio).^33^ It is likely that the high temperature is enhancing this stress response, contributing to, and potentially increasing, the stabilization of tissues during desiccation and survival in air-dry storage.^13^

The long-term conservation of germplasm comes at a cost and, as genebanks largely rely on public funding, managers are under pressure to reduce their expenditure by optimizing and increasing the efficiency of their management procedures. The frequency of both viability monitoring and regeneration, which incurs the greatest costs, can be reduced by ensuring seeds are at their maximum possible longevity when placed into storage.^34^ Therefore, improving the storage longevity of seeds has the potential to reduce the number of accessions regenerated each year, but only if the quantity of seeds stored is sufficient to provide enough for use before viability drops below the 85% standard, that is, a higher quality seed lot calls for a larger sample to be stored.^35,36^

To conclude, the benefits, originally shown in rice, of an initial period of high-temperature drying on subsequent seed longevity have now been observed in an independent study on soya bean and perhaps also for cowpea, although the data are somewhat variable. Although seed storage experiments (as used in this study), where seeds are stored at a high temperature and MC to accelerate the natural aging process, are commonly used in research to compare the longevity between different seed lots (within or between species), there is still a debate as to how comparable these estimates are to those observed under conventional air-dry storage. However, the storage experiments carried out in this study were conducted at an MC and temperature where the effects of the changes in these variables are well defined by the seed viability equations.^24^ Further to this, the seeds were also hermetically sealed inside aluminum foil packets to not only limit any fluctuation in MC but also to restrict the availability of oxygen (which if freely available can lead to an overestimation in longevity [*σ*] compared with that predicted by the viability equations^37^). Therefore, under such conditions, we expect the improvement in longevity in response to initial high-temperature drying to also be apparent in genebank storage.

These results, and those obtained from similar studies, have major implications for *ex situ* conservation as it is possible that other orthodox species may benefit from alternative drying conditions, especially in regard to temperature. Future research should be carried out to determine whether a two-stage drying procedure is beneficial to other economically important crops, especially those with poor storage longevity. In light of further evidence, it may be necessary, at least for those species where the benefits of a two-stage drying regime have been confirmed, that the FAO standards are adapted accordingly.
